# Prevalent and Incident Tuberculosis Are Independent Risk Factors for Mortality among Patients Accessing Antiretroviral Therapy in South Africa

**DOI:** 10.1371/journal.pone.0055824

**Published:** 2013-02-13

**Authors:** Ankur Gupta, Robin Wood, Richard Kaplan, Linda-Gail Bekker, Stephen D. Lawn

**Affiliations:** 1 Department of Clinical Research, Faculty of Infectious and Tropical Diseases, London School of Hygiene and Tropical Medicine, London, United Kingdom; 2 The Desmond Tutu HIV Centre, Institute for Infectious Disease and Molecular Medicine, Faculty of Health Sciences, University of Cape Town, Cape Town, South Africa; National Institute of Allergy and Infectious Diseases, United States of America

## Abstract

**Background:**

Patients with prevalent or incident tuberculosis (TB) in antiretroviral treatment (ART) programmes in sub-Saharan Africa have high mortality risk. However, published data are contradictory as to whether TB is a risk factor for mortality that is independent of CD4 cell counts and other patient characteristics.

**Methods/Findings:**

This observational ART cohort study was based in Cape Town, South Africa. Deaths from all causes were ascertained among patients receiving ART for up to 8 years. TB diagnoses and 4-monthly CD4 cell counts were recorded. Mortality rates were calculated and Poisson regression models were used to calculate incidence rate ratios (IRR) and identify risk factors for mortality. Of 1544 patients starting ART, 464 patients had prevalent TB at baseline and 424 developed incident TB during a median of 5.0 years follow-up. Most TB diagnoses (73.6%) were culture-confirmed. A total of 208 (13.5%) patients died during ART and mortality rates were 8.84 deaths/100 person-years during the first year of ART and decreased to 1.14 deaths/100 person-years after 5 years. In multivariate analyses adjusted for baseline and time-updated risk factors, both prevalent and incident TB were independent risk factors for mortality (IRR 1.7 [95% CI, 1.2–2.3] and 2.7 [95% CI, 1.9–3.8], respectively). Adjusted mortality risks were higher in the first 6 months of ART for those with prevalent TB at baseline (IRR 2.33; 95% CI, 1.5–3.5) and within the 6 months following diagnoses of incident TB (IRR 3.8; 95% CI, 2.6–5.7).

**Conclusions:**

Prevalent TB at baseline and incident TB during ART were strongly associated with increased mortality risk. This effect was time-dependent, suggesting that TB and mortality are likely to be causally related and that TB is not simply an epiphenomenon among highly immunocompromised patients. Strategies to rapidly diagnose, treat and prevent TB prior to and during ART urgently need to be implemented.

## Introduction

HIV-associated tuberculosis (TB) remains a substantial challenge to international public health, accounting for an estimated 1.1 million new TB cases and 0.35 million deaths worldwide in 2010 [1]. A staggering 82% of these cases and 71% of deaths were in sub-Saharan Africa [1]. This burden of disease represents a particular challenge to antiretroviral treatment (ART) programmes in the region as it is concentrated in patients accessing these services [2], [3]. Approximately 5–40% of patients enrolling in ART services have a current TB diagnosis at the time of starting ART [2]–[8]. In addition, there is a high incidence of disease during the initial months of ART, much of which represents prevalent disease present at baseline that was not detected during screening. Long term rates are lowered substantially during ART, but nevertheless remain several fold higher than rates in HIV-uninfected people living in the same communities [9].

Post-mortem studies from sub-Saharan Africa both before and during the ART era have shown diagnosed and undiagnosed TB to be extremely common among hospital in-patients dying with HIV/AIDS, with disseminated disease being observed in 30%–50% of cadavers [10]–[13]. However, it is not entirely clear whether TB is causally related to death, a marker of HIV-associated immunodeficiency that is not reflected by CD4 cell counts or is simply an epiphenomenon among highly immunocompromised patients [14]. TB has been reported as a leading cause of death in ART programmes and yet attributing causes of death is difficult, especially since multiple pathologies are so common. While many studies agree that TB patients in ART programmes have high mortality risk in unadjusted analyses, contradictory findings are reported regarding whether TB is a risk factor for mortality that is independent of the baseline CD4 cell count and other patient characteristics [5]–[8], [15]–[18]. A meta-analysis of studies found no such association but concluded that data were insufficient to draw definite conclusions [19].

Many factors may explain the differences between study findings. Widely used, non-culture-based TB diagnostics perform poorly in this patient population, leading to considerable under-diagnosis and some over-diagnosis. Thus, misclassification within diseased and comparator groups may be important [20]. Most patients with prevalent TB do not start ART until some weeks after commencing TB treatment such that there is substantial survival bias among those who subsequently start ART. Moreover, most studies have simply assessed mortality with respect to baseline patient characteristics and have not accounted for the rapid changes in mortality risk that occur in association with time-updated variables such as CD4 cell counts and plasma viral load [21], [22].

Identification of successful interventions to address the high overall mortality in ART services in sub-Saharan Africa must be based on careful identification of the underlying risk factors and causes [23]. In this study, we therefore sought to assess the association of prevalent and incident TB with mortality risk after controlling for other risk factors, including age, duration of ART and time-updated immunovirological responses to ART. In this cohort in Cape Town, South Africa, culture-based diagnosis of TB is routinely available and so a majority of disease is microbiologically proven. The duration of follow-up was prolonged and time-updated CD4 cell counts and frequent viral load measurements were made during follow-up.

## Methods

### Study Setting

This study was part of ongoing research at the ART service in Gugulethu township, Cape Town, which has previously been described [2], [9], [24]. Patients enrolled consecutively into the ART programme from September 2002 until May 2006 were eligible for the study. Patients were excluded if they did not initiate ART, were non-naïve to ART at enrolment due to transfer in from another service and if aged less than 16 years. All other patients were included in the analysis and data was censored on 1^st^ January 2011, covering a period of over 8 years.

Programmatic criteria for starting ART followed national guidelines, based on the 2002 WHO recommendations, providing free ART for those with a CD4 cell count <200 cells/µL or World Health Organization stage 4 disease [25]. Patients were referred from other clinics to start ART and were routinely reviewed at pre-treatment screening visits, treatment initiation, after 4, 8 and 16 weeks of ART, and at least every 4 months thereafter. Patients had open access to the clinic at all other times. Blood CD4 cell counts and plasma viral load levels were done routinely at baseline and every 4 months. All patients were allocated to community-based therapeutic counsellors for ongoing support, also providing an efficient system for determining outcomes and tracing those who did not attend follow-up appointments. Patients predominantly received efavirenz-based ART but did not receive isoniazid preventive therapy (IPT) as was the practice at that time. All patients received prophylaxis with trimethoprim-sulphamethoxazole before and during ART.

### Tuberculosis Screening, Diagnosis and Definitions

TB screening was conducted at baseline using a symptom-screening questionnaire to identify patients who required further investigation. Investigations available to diagnose TB at baseline or during ART included sputum induction, sputum smear fluorescence microscopy, automated liquid culture of sputum using mycobacterial growth indicator tubes (MGIT 960, Becton Dickinson, Sparks, Maryland, USA), chest radiology, ultrasonography and fine needle lymph node aspiration and cytology. All microbiological specimens were analysed in accredited laboratories, and all positive cultures were speciated by polymerase chain reaction.

‘Past history of TB’ was defined as a diagnosis of TB with completion of anti-tuberculosis treatment before enrolment for ART and was ascertained from referral letters and from questioning patients. ‘Prevalent TB’ was defined as patients taking anti-tuberculosis treatment at the time of starting ART. These were TB diagnoses either made prior to referral for ART or between screening and ART initiation. ‘Incident TB’ was defined as a new clinical episode of TB diagnosed after initiation of ART irrespective of date of symptom onset. Diagnoses of ‘recurrent TB’ were made with reference to any previous episodes of TB. Patients with multiple diagnoses of incident TB had completed a full course of anti-TB treatment with resolution of symptoms between episodes, as ascertained from clinical records.

All cases of TB fulfilled WHO diagnostic criteria for resource-constrained settings with high HIV prevalence [26]. Any culture-negative TB diagnoses were defined by radiological abnormalities consistent with active TB, strong histological or clinical evidence of active TB and a clinician’s decision to treat with a full course of anti-tuberculosis chemotherapy. TB treatment was rifampicin-based and administered via a local network of community clinics providing a directly observed treatment short-course strategy.

### Data Collection and Ethics

Data on baseline characteristics and TB events was obtained from prospectively maintained structured clinical records. Laboratory results were transferred weekly to an electronic database. Patient outcomes during ART were classified as follows: alive and receiving ART, death from any cause, transfer to another ART service, or lost to follow-up (LTFU). LTFU was defined as patients on ART who were over 12 weeks late for a scheduled clinic appointment, failed to return to the ART programme and could not be located by active community-based follow-up. Data for patients LTFU was censored at their last clinic visit. Deaths were ascertained by hospital or medical records, discharge letters or by active community-based follow-up by peer counsellors as previously described [27].

All patients enrolled in the study gave written, informed consent to participate. The collection of data on this study cohort for research purposes was approved by the Research Ethics Committee of the University of Cape Town.

### Data Analysis

Data were analysed using Stata version 11.0 (College Station, Texas, USA). Person-time was accrued from the date of starting ART until death, transfer, LTFU or censoring of observations on 1^st^ January 2011. Chi-squared tests were used for comparing proportions, t-tests for comparing means and Wilcoxon rank-sum tests for comparing medians. All statistical tests were two-sided at α value of 0.05.

Person-time was divided into intervals predefined by the CD4 cell count measurements done at least 4-monthly. Each interval was defined by the CD4 cell count and viral load at the start of the interval, and categorised into CD4 cell count strata and viral load strata. If CD4 cell count or viral load measurements were missing for one or more intervals, a mean of the values for the preceding and subsequent intervals were used (1.6% of intervals). Poisson regression models were used to calculate incidence rate ratios (IRR) with 95% confidence intervals (CI) for time-updated and baseline characteristics. Poisson regression was chosen to allow a description of the effect of ART duration on mortality rates in addition to controlling for it. Cross-tabulation and chi-squared tests were used to assess associations between variables. A multivariate model was constructed using Poisson regression and based on forward selection; *a priori* risk factors (age and duration of ART) and variables identified as being associated with incident TB during the unadjusted analysis were included. Likelihood ratio tests were used to investigate interactions between variables, statistical hypotheses and linear trends.

Several exploratory analyses were also performed to better understand which individuals were at greatest risk of mortality during ART. These models stratified TB diagnoses by recurrent disease, multiple TB diagnoses during ART, culture-confirmed incident TB and by time following TB diagnosis. Poisson regression was used along with aforementioned risk factors and covariates.

## Results

### Patients Enrolled and Follow-up

**Figure 1:**
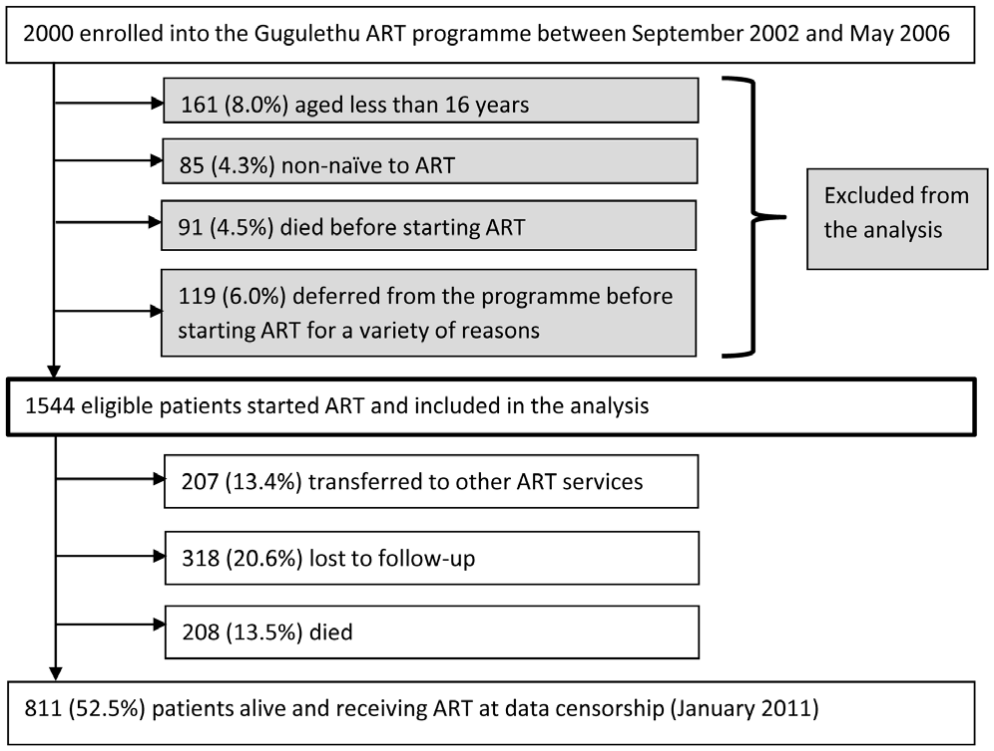
Patient enrolment in the antiretroviral treatment service and outcomes during follow-up.

During the study period, 2000 patients enrolled into the ART programme. A total of 456 were excluded because they were aged less than 16 years (n = 161), non-naïve to ART (n = 85), died before starting ART (n = 91) or deferred from starting ART for a variety of reasons (n = 119) (**Figure 1**). 1544 (77.2%) initiated ART and were included in the analysis. Of these patients, 208 (13.5%) died during ART, 318 (20.6%) were lost to follow-up and 207 (13.4%) were transferred to other ART services. Median follow-up time for patients enrolled in the study was 5.0 years (IQR 2.4–5.4; range 0.0–8.2).

The mean age was 34 years, and the majority of patients were women (70.1%). Overall, patients had advanced immunodeficiency at enrolment with a median baseline CD4 cell count of 98 cells/µL (IQR 48–155). Patients that died were older and more likely to be male have WHO stage 3 or 4 disease, lower baseline CD4 cell counts and higher HIV viral loads (**Table 1**).

**Table 1:** Characteristics for all patients and those alive at data censorship, lost to follow-up and died.

		All patients(n = 1544)	Alive^c^ (n = 811)	Died (n = 208)	Lost to follow-up(n = 318)	p-value^d^
**Mean age (years) [95% CI]**		34 [33.6–34.4]	34.1 [33.6–34.6]	**36.8 [35.5–38.1]**	**32.1 [31.3–33.0]**	<0.001
**Gender**	**Male**	462 (29.9)	236 (29.1)	**85 (40.9)**	**93 (29.3)**	0.006
**WHO stage**	**1&2**	305 (19.8)	184 (22.7)	**14 (6.7)**	**67 (21.1)**	
	**3**	861 (55.8)	451 (55.6)	**108 (51.9)**	**186 (58.5)**	<0.001
	**4**	378 (24.5)	176 (21.7)	**86 (41.4)**	**65 (20.4)**	
**Baseline CD4 cell count** **(cells/µL)**	**Median [IQR]**	98 [48–155]	102 [51–158]	**75 [22–136]**	**105 [53–168]**	<0.001
**Baseline viral load** **(log_10_ copies/ml)**	**Median [IQR]**	4.87 [4.46–5.26]	4.84 [4.45–5.25]	**5.04 [4.63–2.58]**	**4.82 [4.37–5.79]**	<0.001
**Past history of TB** a		725 (47.0)	368 (45.4)	**131 (63.0)**	**133 (41.8)**	<0.001
**Prevalent TB** b		464 (30.0)	212 (26.1)	**82 (39.4)**	**103 (32.4)**	0.099
**Incident TB during ART**		424 (27.5)	233 (28.7)	**59 (28.4)**	**83 (26.1)**	0.489

Data are numbers (%) unless otherwise stated. ART, antiretroviral therapy; TB, tuberculosis; IQR, interquartile range.

a includes all TB episodes where anti-TB treatment was completed prior to ART enrolment.

b TB episodes where patients are taking anti-TB treatment at ART initiation.

c alive at data censorship on 1^st^ January 2011.

d calculated comparing patients that died with those LTFU.

### TB Diagnoses

Almost half of patients (47.0%) had a past history of TB and had completed a course of anti-tuberculosis treatment before starting ART (**Table 1**). At the time of ART initiation, 30% (n = 464) of patients were receiving anti-tuberculosis treatment (prevalent TB). Diagnoses of incident TB were made among 424 (27.5%) patients during ART. In total there were 484 diagnoses of incident TB, with 49 patients having 2 or more diagnoses of incident TB. Overall, 73.6% of TB diagnoses were confirmed by culture.

### Mortality Rates

208 deaths occurred during ART with an overall mortality rate of 3.2 deaths/100 person-years (PY) (95% CI 2.79–3.66). A majority of deaths (n = 124; 59.6%) occurred during the first year of ART with a rate of 8.84 deaths/100 PY (95% CI 7.41–10.54). The mortality rate decreased 4-fold in the second year of ART (n = 26, 2.07 deaths/100 PY, 95% CI 7.41–10.54). Thereafter, rates during years 2–3, 3–4, 4–5 and >5 years of ART decreased to 1.67 (n = 19, 95% CI 1.06–2.62), 1.86 (n = 19, 95% CI 1.18–2.91), 1.22 (n = 11, 95% CI 068–2.21) and 1.14 (n = 9, 95% CI 0.59–2.19) deaths/100 PY respectively.

Mortality rates during ART were substantially greater among patients with prevalent TB at baseline compared to those who were TB-free (4.84 versus 2.62 deaths/100 PY; p<0.001). Similarly, mortality rates were almost double during person-time accrued following incident TB compared to person-time without incident TB (5.02 versus 2.79 deaths/100 PY; p<0.001). Mortality rates calculated using only culture-confirmed incident TB cases were similar to rates considering all TB diagnoses (4.53 deaths/100 PY, 95% CI 3.45–6.12; p = 0.61).

Next, mortality rates stratified by time-updated CD4 cell counts were calculated for the overall cohort and for person-time accrued after diagnoses of incident TB (**Table 2**). Rates were highest (22.87 deaths/100 PYs (95% CI 18.80–27.83) during person-time accrued with CD4 cells counts <100 cells/µL and decreased to 0.72 deaths/100 PYs (95% CI 0.42–1.20) during person-time accrued with CD4 cell counts >500 cells/µL. Mortality rates following a diagnosis of incident TB were also strongly associated with time-updated CD4 cell counts (**Table 2**). Rates were 34.7 deaths/100 PYs (95% CI 23.4–51.3) during person-time accrued with CD4 cells counts <100 cells/µL, decreasing to 8.0 deaths/100 PYs (95% CI 4.6–14.1) and 1.1 deaths/100 PYs (95% CI 0.4–2.9) with CD4 cells counts 101–200 and >500 cells/µL, respectively.

**Table 2:** Overall mortality rates and mortality rates following incident TB during ART by CD4 cell strata.

Updated CD4 cell countstrata (cells/µL)	Overalldeaths	Overall PYat risk	Overall mortality rateper 100 PY (95% CI)	Deaths followingincident TB^a^	PY at risk followingincident TB	Mortality rate per100 PY^a^ (95% CI)
**≤100**	100	437	22.9 (18.8–27.8)	25	72	34.7 (23.4–51.3)
**101–200**	47	982	4.8 (3.6–6.4)	12	149	8.0 (4.6–14.1)
**201–300**	24	1171	2.1 (1.4–3.1)	10	198	5.0 (2.7–9.4)
**301–400**	17	1138	1.5 (0.9–2.4)	4	208	1.9 (0.7–5.1)
**401–500**	7	968	0.7 (0.3–1.5)	4	183	2.2 (0.8–5.8)
**>500**	13	1810	0.7 (0.4–1.2)	4	365	1.1 (0.4–2.9)

ART, antiretroviral therapy; TB, tuberculosis PY, person-years; CI, confidence intervals.

a During person-time accrued following a diagnosis of incident TB.

### Risk Factors for Mortality

In unadjusted analyses, mortality was strongly associated with baseline characteristics including lower CD4 cell counts, higher viral load, increasing age, male gender, WHO stage 3 and 4 disease, past history of TB before ART initiation and prevalent TB at ART initiation (**Table 3**). Shorter duration of ART, time-updated viral load counts >1000 copies/ml, lower time-updated CD4 cell counts and incident TB during ART were also strongly associated with mortality. Person-time accrued at CD4 cell counts <100 cells/µL had a 30 times greater risk of mortality compared to person-time at CD4 cell counts >500 cells/µL (IRR 31.84; 95% CI 17.9–56.7; p<0.001).

**Table 3:** Unadjusted and multivariate analyses of risk factors for mortality during ART.

		Unadjusted analysis			Multivariate analysis		
		IRR	95% CI	p-value	IRR	95% CI	p-value
**Age at enrolment** a		1.04	1.02–1.06	<0.001	1.04	1.02–1.06	<0.001
**Gender**	**F**	1			1		
	**M**	1.73	1.31–2.28	<0.001	1.09	0.80–1.47	0.571
**WHO stage at enrolment**	**1 & 2**	1			1		
	**3**	2.88	1.65–5.02	<0.001	1.77	0.97–3.19	<0.001
	**4**	5.50	3.13–9.67		3.41	1.87–6.24	
**Baseline CD4 cell count (cells/µl)**	**≥150**	1			1		
	**100–149**	1.08	0.69–1.69		0.83	0.57–1.21	
	**50–99**	1.30	0.86–1.97	<0.001	1.52	0.96–2.43	0.020
	**<50**	2.04	1.39–2.99		1.67	1.04–2.65	
**Baseline viral load (log_10_ copies/ml)**	**<5**	1			1		
	**≥5**	1.64	1.24–2.17	0.001	1.34	1.01–1.79	0.040
**Duration of ART (months)**	**0–12**	1			1		
	**12–24**	0.23	0.15–0.36		0.61	0.38–0.98	
	**24–36**	0.19	0.12–0.31		0.62	0.36–1.07	
	**36–48**	0.21	0.13–0.34	<0.001	0.66	0.37–1.16	0.123
	**48–60**	0.14	0.07–0.26		0.51	0.26–1.02	
	**>60**	0.13	0.07–0.25		0.45	0.21–0.96	
**Updated CD4 cell count (cells/µl)** d	**>500**	1			1		
	**401–500**	1.00	0.40–2.52		0.93	0.37–2.35	
	**301–400**	2.08	1.01–4.28		1.77	0.84–3.71	
	**201–300**	2.85	1.45–5.60	<0.001	1.87	0.90–3.87	<0.001
	**101–200**	6.66	3.60–12.31		2.98	1.44–6.20	
	**0–100**	31.84	17.87–56.74		10.41	4.69–23.14	
**Updated viral load (copies/ml)** d	**<1000**	1			1		
	**≥1000**	8.46	6.41–11.17	<0.001	2.74	1.86–4.04	<0.001
**Past History of TB at baseline** b	**No**	1					
	**Yes**	1.87	1.41–2.47	<0.001			
**Prevalent TB at baseline** c	**No**	1			1		
	**Yes**	1.85	1.40–2.44	<0.001	1.67	1.23–2.25	0.001
**Incident TB**	**No**	1			1		
	**Yes**	1.80	1.33–2.43	<0.001	2.70	1.91–3.81	<0.001

ART, antiretroviral therapy; TB, tuberculosis; IRR, incident rate ratio; CI, confidence interval. All p-values were calculated using the likelihood ratio test.

a Age is included as continuous variable, the IRR represents a 1 year increase in age.

b includes all TB episodes where anti-TB treatment was completed prior to ART enrolment.

c TB episodes where patients are taking anti-TB treatment at ART initiation.

d CD4 cell counts and viral load are time-updated variables using serial measurements taken during ART.

**Figure 2:**
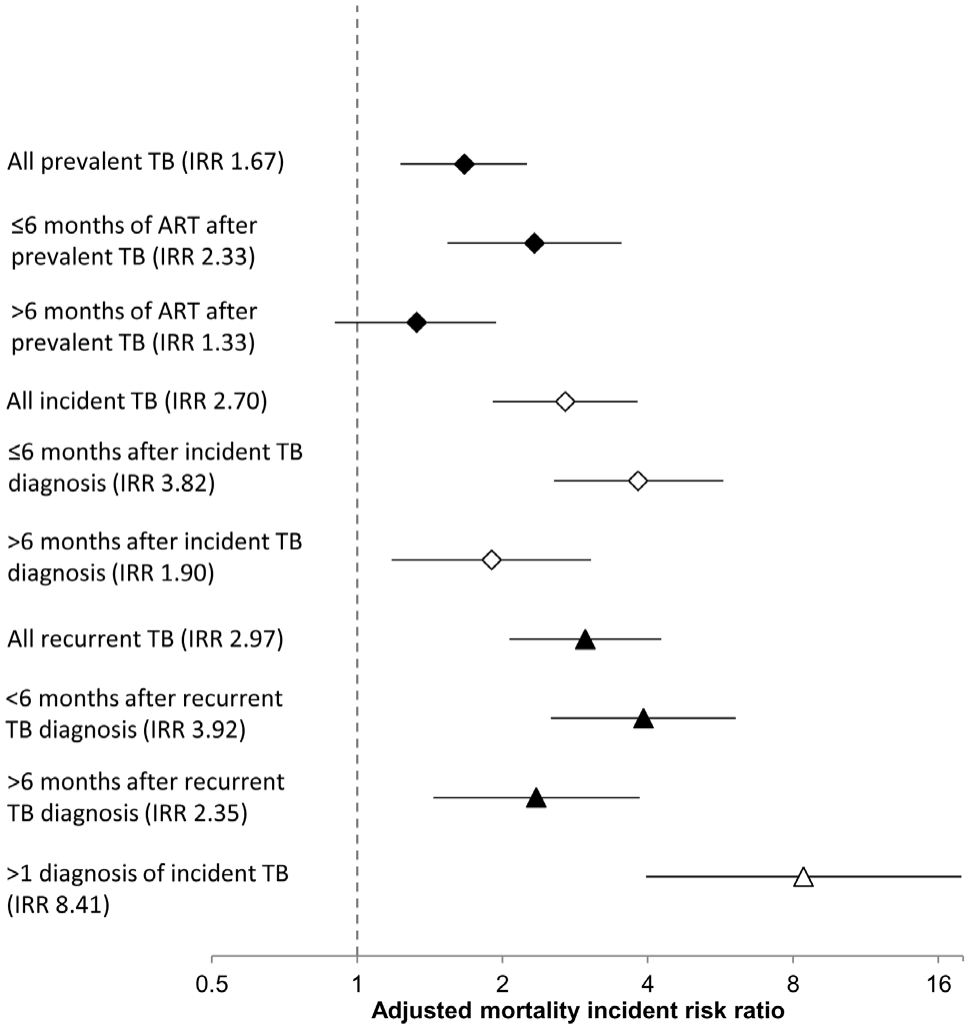
Mortality incident risk ratios (IRR) associated with person-time accrued following prevalent (⧫) and incident (⋄) tuberculosis (TB) compared to person-time with no TB diagnosis, adjusted for time-updated CD4 cell count and HIV viral load, and other time-updated and baseline risk factors, stratified by timing after TB diagnosis, recurrent TB including all previous TB diagnoses (▴) or multiple diagnoses of incident TB (▵).

Past history of TB was excluded from the multivariate analysis due to a strong association with WHO stage, since pulmonary TB is a stage 3 defining condition and extrapulmonary TB is a stage 4 defining condition. In multivariate analysis, time-updated CD4 cell counts remained the strongest risk factor for mortality (**Table 3**). Prevalent TB at ART initiation was associated with a 1.7-fold increased mortality risk (IRR 1.67, 95% CI 1.23–2.25, p = 0.001). Mortality risk during the first 6 months of ART in those with prevalent TB at baseline was 2.3-fold higher (IRR 2.33, 95% CI 1.54–3.53) than that among those who did not have prevalent TB. Mortality risk >6 months after starting ART was not significantly increased among those with diagnoses of prevalent TB compared to those without (IRR 1.33, 95% CI 0.90–2.25, p = 0.141) (**Figure 2**).

After adjustment for other baseline and time-updated risk factors, incident TB during ART was associated with a 2.7-fold greater mortality risk compared to person-time with no TB diagnoses (IRR 2.70, 95% CI 1.91–3.81, p<0.001). Incident TB also showed a time-dependent effect on mortality risk in multivariate models separately assessing person-time ≤6 months and >6 months after a TB diagnosis. Compared to person-time without incident TB, mortality risk ≤6 months after a TB diagnosis was almost 4-fold higher (IRR 3.82, 95% CI 2.56–5.74). Person-time >6 months after a diagnosis of incident TB was associated with a smaller increase in mortality risk (IRR 1.90, 95% CI 1.18–3.05) (**Figure 2**).

The adjusted mortality risk associated with diagnoses of incident TB that were recurrent (IRR 2.97, 95% CI 2.07–4.27) was higher than that associated with incident TB that was non-recurrent (IRR 1.72, 95% CI 0.82–3.61) and again this risk was time-dependent (**Figure 2**). Moreover, person-time following multiple diagnoses (2 or more) of incident TB was associated with a more than 8-fold increase in adjusted mortality risk compared to person-time without a TB diagnoses (IRR 8.41, 95% CI 3.97–17.86) (**Figure 2**). In the multivariate analysis, gender and duration of ART were no longer associated with mortality.

### Loss to Follow-up

Among patients initiating ART, 318 were LTFU. Baseline characteristics differed significantly between patients who died and those that were LTFU (**Table 1**). Compared to those who died, patients who were lost to follow-up were younger, more likely to be female and have WHO stage 1 or 2 disease, higher baseline CD4 cell counts, lower HIV viral loads and fewer had a past history of TB. A multivariate model constructed assuming all patients LTFU were unascertained deaths still found prevalent TB (IRR 1.49, 95% CI 1.22–1.81) and incident TB (IRR 2.07, 95% CI 1.68–2.56) to be independent risk factors for mortality.

## Discussion

This study examined the impact of TB on mortality in an ART cohort during long-term follow-up. The key findings are that, after adjustment for other risk factors including time-updated CD4 cell counts, person-time on ART in patients with prevalent and incident TB remained independently associated with a 1.7 and 2.7-fold greater mortality risk, respectively, compared to person-time in patients on ART without TB. This finding strongly supports the view that TB is an important risk factor associated with high mortality in ART programmes in sub-Saharan Africa despite some studies suggesting the contrary [19]. There was also a time-dependent relationship between TB and mortality risk, with much higher adjusted mortality risk within the first 6 months of ART in patients with baseline prevalent TB (IRR 2.3) and in the 6 months following diagnoses of incident TB (IRR 3.8). These findings are similar to another study in South Africa which showed mortality risk associated with TB diagnoses waned over time [16]. The strong time-dependent association between TB episodes and increased mortality risk suggests that TB and mortality are causally related, and that TB is not simply an epiphenomenon among this highly immunocompromised patient population. Effective strategies to diagnose, rapidly treat and prevent TB both prior to and during ART urgently need to be implemented.

The huge burden of TB in this cohort is higher than in ART services in other countries in sub-Saharan Africa but is similar to others in South Africa [5], [17], [28]–[30]. The 2–3 fold increase in mortality risk associated with such a common risk factor represents a major challenge to ART programmes. The temporal distribution of mortality risk and TB diagnosis further supports the association between TB and death during ART. This could be hypothesized as being a direct association with TB itself, or the result of other associated factors such as drug co-toxicity, poor treatment adherence due to high pill burden or TB immune reconstitution disease. However, excellent immunovirological responses to efavirenz-based ART have been documented in this cohort, drug co-toxicity with this regimen is very low, and deaths from TB immune reconstitution disease are infrequent (a recent meta-analysis found only 3.2% of those with TB-associated immune reconstitution disease died) [2], [31]–[33]. Thus, the most plausible explanation is that mortality was directly associated with active TB disease. We were unable to estimate the prevalence and impact of multi-drug resistance (MDR). Another more recent study in this cohort has found MDR-TB in 5% of isolates and this may well be another important factor [34].

A huge burden of TB was diagnosed under routine programme conditions in this cohort, as previously described [2]. However, more recent studies in this cohort have found that in patients with no baseline TB diagnosis, systematic intensive culture-based screening detects sputum culture-positive TB in 18% to 25% of cases [34]–[36]. These findings support those of post-mortem studies conducted in sub-Saharan Africa which have revealed a huge burden of unascertained TB in HIV-positive patients [10]–[13]. Together these data suggest there remains a strong likelihood that additional cases of TB remained unascertained in patients that died during our study. Thus, we may have underestimated the association between TB and mortality. Moreover, this study did not account for deaths associated with prevalent TB that occurred before ART was started. This may account for the weaker association between mortality risk and prevalent TB compared to that between mortality and incident TB. Another potential reason for this difference is that, in addition to prevalent TB, multiple other opportunistic infections occur at low CD4 cell counts and may ‘compete’ as causes of death whereas during person-time at high CD4 cell counts with long-term ART opportunistic infections other than incident TB are uncommon.

Early detection of TB through active screening and rapid initiation of treatment in diagnosed cases may potentially reduce mortality [23]. New rapid diagnostics have the potential to greatly expedite TB diagnosis compared to culture-based diagnosis that was routinely used in this cohort. The Xpert MTB/RIF (Cepheid Inc, Sunnyvale, CA, USA) is a simplified rapid molecular assay and the Determine TB-LAM Ag (Alere, Waltham, MA, USA) is a simple lateral-flow point-of-care assay that detects mycobacterial lipoarabinomannan (LAM) in urine samples [34], [35]. These both offer the prospect of rapidly diagnosing TB, even in those with the most advanced immunodeficiency and highest mortality risk [37], [38]. Prospective intervention studies are needed to determine the impact of these assays on patient outcomes.

Once TB is diagnosed, patients need optimised case management, including well integrated and patient-centred services providing TB treatment, ART started in the course of TB treatment (with optimal timing now defined by randomized controlled trials) and trimethoprim-sulphamethoxazole prophylaxis [23], [39]. Preventive measures are also required for those who do not have TB. Prevalent TB can be prevented by initiation of ART earlier in the course of HIV infection, thereby reducing time accrued at lower CD4 cell counts and subsequent TB risk [22], [40]. IPT can prevent TB prior to ART, and evidence from observational studies also suggests an additive effect when combined with ART [41], [42]. Improving ART adherence and access to second-line ART regimens should prevent TB and reduce mortality by optimising immune recovery [43], [44]. Further data on the impact of drug resistance will also help inform interventions and investments in drug-susceptibility testing and new therapeutics.

Strengths of this study include the long median duration of follow-up and high proportion of culture-confirmed TB diagnoses. Routine monitoring of CD4 cell count and viral load 4-monthly provides accurate information on time-updated immune response. The observational design of this study has inherent limitations, including the inability to establish a direct causal relationship between TB and mortality, but the routine programmatic conditions may improve the generalisability of the findings.

The distinction between prevalent and incident TB is blurred and some prevalent disease missed at baseline screening may have been classified as incident disease. Data on other incident AIDS or non-AIDS defining illnesses were not available. If these occurred around the time of TB diagnosis and were associated with death, there may be an overestimation of the association between mortality and tuberculosis. Patients classified as LTFU may represent unascertained mortality and unascertained TB. Although some studies have found high rates of mortality amongst patients LTFU, earlier data from this cohort suggested relatively low mortality amongst those LTFU [27], [45]. Furthermore, the present sensitivity analyses showed that even if all patients LTFU were considered to be unascertained deaths, the association between TB and mortality remained. This study was unable to account for some important biological and social risk factors for mortality, especially opportunistic infections with high case fatality ratios such as cryptococcal meningitis and systemic sepsis, which may have also caused an overestimation of the association between TB and mortality [46]. However, most opportunistic infections are strongly associated with CD4 cell count which was adjusted for.

Data on anaemia and body mass index (BMI), both strong independent predictors of mortality [14], were not accounted for in our multivariate model. This may be appropriate as both low BMI and anaemia are strongly associated with TB and mycobacterial load, and thus are very likely to lie on the causal pathway [37], [47]. Cause of death could not be definitively determined for most patients in this study. Few data exist on pathologically-proven causes of ART-associated mortality from sub-Saharan Africa. Such data are needed to further investigate the relationship between TB and mortality in these settings.

In conclusion, our study shows a strong association between prevalent and incident TB and mortality during ART. Strategies to prevent TB prior to and during ART are needed, as are diagnostic algorithms and new tools to rapidly identify cases and initiate treatment. Such interventions are likely to represent key interventions that are desperately needed to address the high mortality rates in ART programmes in sub-Saharan Africa.
